# An Evaluation of the Non-Neutrality of Money

**DOI:** 10.1371/journal.pone.0145710

**Published:** 2016-03-02

**Authors:** Tito Belchior Silva Moreira, Benjamin Miranda Tabak, Mario Jorge Mendonça, Adolfo Sachsida

**Affiliations:** 1 Department of economics, Catholic University of Brasilia, Brasília, Brazil; 2 Department of economics, Catholic University of Brasilia, Brasília, Brazil; 3 Department of economics, Institute for Applied Economic Research, Rio de Janeiro, Brazil; 4 Department of economics, Institute for Applied Economic Research, Brasília, Brazil; Uppsala University, SWEDEN

## Abstract

This paper evaluates the effect of a change in the quantity of money on relative prices in the U.S. economy based on quarterly time-series for the period of 1959 to 2013. We also estimate the implication of a change in relative prices on the rate of inflation and macroeconomic variables. The empirical results indicate that the change of money supply not only affects relative prices but also affects the inflation rate and real variables, such as investment, natural rate of unemployment and potential GDP, through the change in relative prices. The relevant finding of our study is that money is not neutral in a non-traditional sense because a change in the money supply disturbs relative prices and, consequently, the allocation of resources in the economy. This finding has serious implications that must be considered in the transmission mechanisms of monetary policy.

## Introduction

Economists in general agree with the idea that the observed variations in the nominal price are related to changes in the quantity of money. The history of hyperinflation corroborates this view [1,2]. In this context, [3] highlight some interesting facts in a cross-section study regarding to the ability of changes in money growth to affect inflation or output change. This study applies different concepts of money supply. The correlations that they compute reveal some interesting points. In the long run (a) there is a high correlation between the rate of money supply and the rate of inflation; (b) there is no correlation between the growth rate of money and real output; and (c) there is no correlation between inflation and real output. In a certain way, these findings are in accordance with what [4] predicted: changes in the money supply cannot disturb real variables in the long run, but only in the short run. This means money is neutral in the long run.

However, there are not enough studies regarding the specific role played by the change in the quantity of money on the structure of relative prices. It can be the case that even when money is neutral in the traditional approach of the quantity theory of money (QTM), changes in the money supply can modify relative prices in the economy.

In other words, despite the macroeconomic effect considering the general price level, one cannot ignore the possibility that there exist microeconomic implications derived from a change in the money supply even in long run. In this sense, the traditional approach of QTM failed to establish the basis for microeconomic studies of monetary economy. Although some economists [4,5,6,7] have already developed works in monetary economics with microeconomic fundamentals, there are still some relevant aspects that an aggregate analysis cannot contemplate.

The equilibrium business cycle theories of Robert E. Lucas and others have opened new theoretical approaches, and among these, Hayek’s theory has reappeared. In his model, [8] shows the trade cycle as an outcome of rational reactions to monetary expansion and subsequent distortions of the price structure. This approach of the crisis, as an inevitable process of reequilibration, provoked much debate in the 1930s [9]. In this context, [10] show a critical evaluation of Hayek’s monetary theory and policy.

There are some elucidative discussions on how the credit policy pursued by a central monetary authority can be a source of economy-wide distortions in the intertemporal allocation of resources and hence an important cause of business cycles based on the canonical Austrian Business Cycle Theory (ABCT) [11,12,13,14].

Furthermore, there are several stylized facts concerning the US time structure of production business cycle during the 2002 through 2009, which are consistent with ABCT [15]. The time-structure is characterized empirically using industry-level input-output data. Specifically industry’s total output requirement is used as a metric for roundaboutness. The author finds that the time structure of production lengthened following the FED’s 2002 expansionary deviation from the Taylor rule and, after that, contracted during the Great recession. See also [16,17] for a discussion on the Austrian Business cycle theory.

In the same vein, [18] show empirical evidence of the Mises-Hayek theory of the trade cycle, whereas [19] and [20] provide an interesting discussion on the great depression.

In the context of procyclical leverage and banking catastrophes, [21] have studied the mechanisms by which a financial crisis spreads. The authors highlight the asymmetric reaction of balance sheet to positive and negative shocks, the possibility of discontinuous asset price declines, and the path dependence of portfolio adjustments in response to changing beliefs.

It is noteworthy that [4] and [22,23] used the term “natural rate of unemployment” to express the idea that the level of unemployment could not be determined by monetary policy because it would result only from forces operating in the real economy. The two authors’ basic idea is that economic policy could not be used to determine the unemployment rate of the economy in the long run, which is consistent with the idea of the neutrality of money. This concept was built in the 1960s to accommodate the tradeoff between inflation and unemployment.

Later [24,25] use a general equilibrium framework with a rational expectations hypothesis to model the natural rate of unemployment and to demonstrate the neutrality of money. In this paper, we assess whether monetary policy through changes in relative prices affects not only the natural rate of unemployment but also the growth rate of potential output. If this is the case, we can reject the notion of the neutrality of money in a non-traditional sense because a change in the money supply disturbs relative prices and, consequently, the allocation of resources in the economy.

The general price level is a statistical average of the prices in an economy. Therefore, it is difficult o assess changes in the structure of relative prices, if it really exists, and its consequence for resource allocation based on this concept.

It is reasonable to accept the hypothesis that if the management of monetary policy is smooth it does not produce any relevant changes in relative prices. There are other situations where conducting monetary policy can generate relevant effects on the allocation of resources. In our view, this happened during the U. S. economic crisis of 2008, for example, where the Federal Reserve Bank kept the federal funds rate low for a long time and the government promoted regulatory policies to finance residential mortgages for low and middle-income people. For some economists the conjugation of these two facts resulted in a speculative bubble in the credit market, which can be perceived as responsible for the crisis [26,27].

Using quarterly data from 1959:2 to 2013:02 we perform an econometric analysis to evaluate the follow issues: i) the direct effect of the change in the money supply on the change in relative prices; ii) the indirect impact of the change of money supply on the inflation rate; iii) the indirect impact of the change of money supply on the variation of real investment; iv) the indirect impact of the change of money supply on the natural rate of unemployment; and v) the indirect impact of the change of money supply on the change in the real potential GDP.

Our database allows plenty of distinct relative prices to be evaluated. We opt for the ratio between the producer price index and the consumer price index to represent the relative price variable. As we show in section 3, one can estimate how much a change in the money supply can distort relative prices.

One of the postulates of the quantity theory of money is the dichotomy between relative prices and absolute prices, which is guaranteed if changes in relative prices are explained by changes in real variables such as GDP and employment, while absolute price movements are explained by changes in the money supply. This dichotomy means that given the supply of money, the velocity of money and the level of trade in goods, changes induced by a real shock in the relative prices produce compensatory changes in other relative prices, so that the absolute level of prices remain unchanged [28]. However, in our view, nothing guarantees that such changes are offset so that the general price level remains unchanged.

The econometric results display evidence that a change in the money supply affects the relative prices. This result does not corroborate the assumption that changes in relative prices only occur due to changes in real variables such as unemployment rate, technological shocks, etc. Moreover, a change in relative prices, resulting from changes in the quantity of money, must be considered when analyzing monetary policy transmission mechanisms because they indirectly affect real economic variables such as investment, natural rate of unemployment and potential output. In sum, we find empirical evidence that money is not neutral in a non-traditional sense because a change in the money supply disturbs relative prices and, consequently, the allocation of resources in the economy.

## Revising the Concept of the Neutrality of Money

The idea behind the neutrality of money is strongly related with the quantity theory of money that was made popular by [29] based on the famous identity, *MV = PY*. This identity states that the price level (*P*) varies directly with the quantity of money (*M*) considering that the velocity (*V*) of circulation of money and the volume of transactions (*Y*) of final goods and services do not change. In this context, [24] published one of the most famous articles on neutrality of money that relates monetary shocks to relative and absolute prices. It shows that monetary shocks have real effects in the short term because agents have information asymmetry and they cannot distinguish whether the change in prices is relative or absolute. The author concludes that in the long run, however, money is neutral.

The majority of theories that try to model money neutrality are in certain way connected with this theory. Of course, as proposed by many economists, a given change in the money supply has an effect on real variables during a transition period, that is, until the price level adjusts completely in a new steady-state equilibrium. Unfortunately, this type of analysis does not take into consideration the microeconomic aspects involved in this process. First, it considers the monetary issues in a secluded bay where the marginal utility, value and prices are not connected. The quantity theory of money developed its concepts through economic aggregates such as general price level, velocity of money and domestic output.

The quantity theory of money assumes that changes in absolute prices are only assigned to monetary causes in the long run and that changes in relative prices are caused only by changes in real variables. In this context, what if a change in relative prices could be caused in part by changes in the money supply? What would be the possible implications of this result? A possible implication would be the non-neutrality of money, even in the long run, and that changes in relative prices should be recognized as a transmission channel of monetary policy. One of the first economists who perceived this point was Ludwig von Mises. The economists [30,31,32] realized that it was necessary to extend the application of the subjectivist theory of value developed by [33] in the scope of monetary economics. [30,31,32] showed that monetary expansions promoted by government with “new money” affect the structure of capital and the price of different markets unequally. Thus, a change in quantity of money is able to cause a change in relative prices. This implication, which refutes the neutrality of money, was further developed by [34,35].

Following this theory, a change in the quantity of money can change relative prices and, consequently, modify price signals. Here the rates of return of the various combinations of capital are altered. This new rearrangement of relative prices of goods and services will differ from those determined by the fundamentals. The new price arrangement points to an increase in the profits of certain firms at the expense of others. What type of monetary expansion can recurrently produce changes in relative prices? In accordance with [30,31,32], there is a classical case in which growth in the money supply distorts relative prices and, consequently, resource allocation. This happens when growth in the money supply is related to the expansion of bank credit not derived from the market but from the injection of new money by the monetary authority. This increase in the money supply increases the availability of loanable funds leading to a fall in interest rates and, in turn, the promotion of investment. In this case, the increase in lending is not matched by growth in the flow of production factors available that can be used to fit this increased demand for new investments. For [30,31,32] this is the main cause of the business cycle.

Another instance in which we note a harmful implication of monetary expansion is the case of monetary inflation. The additional amount of money that enters the economy is not distributed equally to all individuals. The first beneficiaries receive this new quantity of money, which allows them to offer more money in the market in exchange for goods and services they wish to purchase. This additional amount of money in the market places pressure on prices and wages. However, not all prices and wages rise; increases only take place in those sectors that first received the new money in exchange for their goods and services. In addition, even if prices and wages rise, they do not rise at the same proportion. For example, if additional money is spent on public servants’ payroll, only the prices of some goods and only the salaries of some types of work will rise (i.e., prices and wages related to the public sector), while prices in other areas will remain unchanged or may even drop temporarily. They may fall in the market because there are now some groups of individuals whose income has not increased but who are now forced to pay more for the same goods that were consumed earlier. By being forced to pay more for certain goods and services, they begin to consume less than other areas, which can result in a temporary price drop.

Thus, changes in prices due to inflation only start with some goods and services. This process then spreads slowly from one group to another. It takes time until the additional amount of money has permeated the entire economy and has exhausted all possibilities of price change. Even at the end of the process, however, various goods and services are not affected to the same degree. The process of gradual currency depreciation resulting from increased money supply changes the income and wealth of different social groups. The process of depreciation will occur while the additional amount of money does not exhaust all its possibilities to influence prices. The process of accelerating inflation severely and unequally penalizes different social groups. The end result of a long inflationary process is a new economic order resulting in dispersion of wealth and income.

In short, the social and economic consequences of a change in a currency’s purchasing power are twofold. First, because money is the measure by which future payments are made, the relationship between creditors and debtors changes. Second, changes in purchasing power do not affect all prices and wages at the same time and in the same degree. There is a redistribution of wealth and income between different social groups. In our view, economic theory generally does not pay much attention to this issue. In the following section, we present the model that we use to verify whether in fact there exists some effect on relative prices as a consequence of a change in money supply.

## Theoretical Model and Econometric Issues

### Theoretical Model

In this section we introduce the model we apply to check if a change in the quantity of money really produces effects associated with a change in relative prices and, consequently, if this change in relative prices produces an impact on real variables and the inflation rate. For that, we estimate a system of two regressions. The first one correlates relative prices to a change in the quantity of money, and the second one correlates the inflation rate and some real variables to change in relatives prices. This system can be described in the following way. We estimate the first equation as

$$\begin{array}{l} \Delta {\left(P_{i}/P_{j}\;\right)}_{t}\;=\beta_{0}+\sum_{i}^{n}\alpha_{i}\Delta {\left(P_{i}/P_{j}\right)}_{t-i}+\beta_{1}\Delta M_{t}+\beta_{2}\Delta Y_{t}+\\ +\beta_{3}D(60,70)+\beta_{4}D(60,70)*\Delta M_{t}+v_{t} \end{array}$$ (1)

where *n* is the number of lags of the dependent variable, *M*_*t*_ is the change in the money supply, *Y*_*t*_ is the change in the real GDP and (*P*_*i*_/*P*_*j*_)_*t*_ identifies the change in relative prices. We follow the [36] approach and use a dummy for the period 1960–1970, *D*(60,70) and also an interactive variable that shows the impact of changes in the money supply during the period of greater tolerance for inflation (*D*(60,70)*(*M*)).We define the growth rate as follows:(*Log*(*M*2_*t*_/*M*2_*t*−1_)).

The parameter *α* in the autoregressive components tries to capture the inertia in the dynamics of the dependent variable. The basic hypothesis behind this equation is that changes in relative prices result from changes in the quantity of money and from the real sector of the economy that is represented in Eq (1) by the change of real output. The effect on relative prices derived from other variables, for instance the interest rate, occurs through a change in the money supply as a consequence of a change in these variables. We use *M2* to represent the money supply.

The second equation of the system is

$$V_{t}=\delta_{0}+\sum_{i}^{n}\gamma_{i}V_{t-i}\;+\delta_{1}\Delta {\left(P_{i}/P_{j}\;\right)}_{t}\;+\delta_{2}\Delta M_{t}+\delta_{3}(D60,70)*\Delta M_{t}+u_{t}.$$ (2)

Eq 2 estimates the effect of the change in the relative prices on the change of variable *V*_*t*_. This equation also considers a possible inertial effect of the variable of interest. In Eq (2), we represent the dependent variable *V* with the following variables: inflation rate (*π*), variation of real investment(*I*), variation of potential output $(\tilde{Y})\;$ and the natural rate of unemployment(*U*). Thus, we have four systems of simultaneous equations to estimate. We evaluate the indirect effect of the change in the money supply on the inflation rate and on the real variables through relative price, replacing in Eq (2) the dependent variable (*P*_*i*_/*P*_*j*_)_*t*_ of the Eq (1). This effect is given by the term *β*_1_*δ*_1_. We also assume that *c*(*u*_*t*_,*v*_*t*_)≠0. Furthermore, we also consider the direct effect of variation of money on change on variable *V*_*t*_.

Economists agree that management of monetary policy by the Federal Reserve Bank has experienced different regimes during the period 1959:2 to 2013:2. What is more difficult is how to determine these regimes because the operating procedures employed by the Fed have changed overtime. Most economists agree that four different regimes have existed at the Federal Reserve over the past 30 years [37]. These correspond to periods of funds-rate, nonborrowed-reserves, borrowed-reserves and fund-rate operating procedures. Nonborrowed-reserves is a measure of the reserves in the banking system. Non-borrowed reserves represent the numerical difference between total reserves minus funds that have been borrowed from the Fed discount window. The first element of this equation consists of the total reserves held in deposit at the Fed by member banks plus the composite cash in their vaults. The second element is money borrowed by banks through the Fed discount window. Borrowed-reserves are reserves that were obtained by borrowing from the Central Bank.

The period 1972–1979, which dates from the end of Bretton Woods, is usually described as the period in which the Fed followed a federal-funds rate. During the period from 1979 to 1982, the Federal Reserve announced several actions that emphasized management of nonborrowed-reserves. An operating procedure that focused on a reserve quantity was viewed as more consistent with reducing money growth to reduce inflation. From 1982 to 1988, the Fed followed a borrowed-reserves operating procedure. In practice, this regime is similar to a funds-rate operating procedure. Finally, since late 1980s, the Fed has targeted the funds rate directly. In this context, open market operations are conducted throughout each day to control the funds-rate around the target.

Regarding inflation control, for [38] a significant difference exists in the way monetary policy was conducted pre- and post-1979, the year Paul Volker was appointed as Federal Reserve Chairman. They find evidence that the Federal Reserve, not surprisingly, was highly accommodating in the pre-Volker years. In other words, during the Volker-Greenspan era the Fed adopted a proactive stance toward inflation. This study uses quarterly data spanning the period 1960:1 and 1996:4. In the same vein, [36] have argued that the Fed acted in the 1950s and in the post-Volker years with a low tolerance toward inflation. However, the Fed Chairmen were more tolerant of inflation in the 1960s and 1970s. In this sense, [36] also consider two schemes for conducting monetary policy from 1950 onward. The 1950s and again from 1980 onwards are considered periods in which the monetary regimes adopted by the Fed have low tolerance of inflation. On the other hand, the period from 1960 to the late 1970s is associated with a regime characterized by high tolerance of inflation. Indeed, the article by [36] includes an analysis until the period of the Greenspan era. Without loss of generality, we assume the hypothesis that the post Greenspan period was also not tolerant of inflation.

In the current study, we follow the [36] approach. In this context, we use a dummy for the period 1960–1970, *D*(60,70) and also an interactive variable that shows the impact of changes in the money supply during the period of greater tolerance for inflation (*D*(60,70)*(*M*)). The results show that the two monetary regimes are distinct confirming the argument of theses authors.

National account statistics give us many distinct price indices, thus enabling us to compute plenty of relative prices. Nevertheless, we choose only some of them, as without an appropriate economic reason many would not bring relevant appeal or provide any insight. We employ the ratio between producer and consumer indexes as our measure of relative price. This statistic can cover a broader sample of goods. It also gives an idea of the effects on income distribution derived from money expansion. Finally, the government recurrently uses the credit expansion to promote specific sectors. For instance, as we noted in the introduction, the U. S. economic crisis of 2008 happened as a consequence of a speculative bubble in the credit market caused by monetary policy and regulatory policies in the real estate market. If the government targets some specific sector of the economy, the costs of production with capital, materials and wages may rise in this sector.

The theoretical justification to employ this proxy for relative prices is that some theoretical models show that the discount factor in steady-state is equal to the intertemporal rate of substitution between future consumption and current consumption. If one considers the reasonable hypothesis that the producer price is the discounted current price of future consumption, this relationship correlates with the ratio between the consumer price index and the producer price index.

The system composed by Eqs (1) and (2) is subject to some sources of endogeneity. The variable *M*_*t*_, the change in money supply, is endogenously determined by at least three reasons. First, a change in quantity of money has an effect on the relative price, but the relative price can also affect the way in which monetary authority targets monetary aggregates. Second, we use *M2* as the proxy for money supply although there are others measures. Nevertheless, one cannot know which monetary aggregates the Fed really seeks to control. Thus, it can be considered a typical case of error in variables. Finally, when the aim of the operational regime is targeting the funds-rate, the quantity of money is the variable that adjusts to the level necessary to fit this rate. In this context, the money supply is determined by the funds-rate. However, the federal funds rate can target any of the dependent variables of Eq (2). In the following section, we describe the appropriated econometric methodology we apply in dealing with endogeneity.

### Econometric issues

Eqs (1) and (2) define a simultaneous equations model. Due to the endogeneity problem, we apply the generalized method of moments (GMM), which requires the employment of instrumental variables (IV). For the appropriate use of the IV method, the instruments must be "good instruments" to be relevant and valid. This implies that the instruments must be not only correlate with endogenous regressors but also be orthogonal to the disturbance. The following tests are applied to our econometric specification: the test of underidentification [39], the test of overidentifying Sargan-Hansen also known as J-statistic, and the Stock-Yogo test [40] to verify the hypothesis of weakness of instruments.

For an equation to be identified in the IV model both the model order condition (L> = K), where L is the number of instrumental variables and K is the number of regressors, the rank condition must be fulfilled. The latter notes that Qxz = E (X'Z) must be full rank, where X = (X1, X2) = (Endogenous, Exogenous) is the matrix of regressors, while Z = (Z1, Z2) = (excluded, included) is the matrix of instruments, and Z2 = X2. When this does not occur we say that the model is underidentified or unidentified. One can test the rank condition through the Cragg-Donald test [39]. If we cannot reject the null hypothesis then the model is underidentified.

The independence of the instrument with respect to the error term can only be accessed if, and only if, there is an "abundance" of instruments, i.e., if the equation is over-identified. This happens when the order condition is satisfied in inequality: the number of excluded instruments exceeds the endogenous regressors. The Sargan/Hansen test is used to test the hypothesis of over-identification, under the null hypothesis that instruments are valid, i.e., uncorrelated with the disturbance. Even under this hypothesis, the test statistic has a chi-square distribution with L-K overidentifying restrictions.

Instruments that have low explanation of the variation (weak correlation between X and Z) of the endogenous explanatory variable are considered weak instruments. The Stock-Yogo test [40] is calculated based on the F statistic of Cragg-Donald [39]. Under the null hypothesis the estimator is weakly identified in the sense that the observed bias is unacceptably large.

The econometric research on instrumental variables has greatly emphasized the problem of weak instruments[40,41,42]. In this context, [43] notes that the use of instruments can be a serious problem. When instruments are weak, two problems can occur. The first one is bias. Although the IV estimator could be consistent, estimates are always biased for small samples. Second, when the instruments are weak the estimated standard error becomes very small. Thus, the confidence interval is not reliable because the midpoint of this estimator is biased and the confidence interval becomes large. As shown by[44], the problem of weak instruments may happen even if in the first stage the tests are significant at conventional levels (5% or 10%) in a large sample.

Various tests are suggested in the literature to verify the hypothesis of weak instruments. One statistic commonly used is the R2 of the first stage with "included" instruments[45]. Alternatively, this can also be expressed as a joint F test the significance of the excluded instruments Z1. However, when more than one variable is endogenous, this indicator is not valid any more. [46] proposed a statistic named by "partial R2" that captures the correlation between the instruments. When just one regressor is endogenous, the two R2 measures are equivalent. Another rule applied when there is only one endogenous regressor is the value of the F statistic in the first stage. In this case, a value less than 10 indicates the instruments are weak. Alternatively, [40] suggested a test where the null hypothesis is that the bias of the estimator 2SLS is less than a fraction (say 10%) of the OLS estimator. According to[47], the latest approach to the problem of hypothesis testing with weak instruments and one endogenous regressor is the "ratio test conditional likelihood" [42,48]. The test of [42] overcomes the distortions found in conventional tests adjusting critical values according to each sample to generate the appropriated level of significance. Thus, the critical values are conditioned on the data available and are not constant.

Finally, when the variables are not stationary, specific problems arise in conventional inference based on ordinary last squares (OLS) regressions. In this sense, [49] stress the importance of knowing whether similar problems occur in the context of two-stage least squares regressions. Notwithstanding, [50,51] analyzes this issue and concludes that the inference with two-stage least squares estimators using instrumental variables remains valid, even when time series are non-stationary or non-co-integrated. In that context, Hsiao’s conclusions are also valid when GMM is applied.

To take into account the two problems of unknown heteroskedasticity and the serial correlation of the residuals, we use the procedure of Newey and West for all estimated models [52,53]. The authors have proposed a more general covariance estimator that is consistent in the presence of both heteroskedasticity and autocorrelation of an unknown form.

#### Structural VAR (SVAR)

As we pointed out it is likely that *V* and the change in the relative price ratio are probably co-determined. Then we use another methodology in order to check the effect of a change in the relative prices on the variables represented by *V*. Here we apply structural VAR (SVAR) analysis. Good descriptions of structural VAR can be found in [54,55,56,57,58]. The SVAR allows to derive the impulse response functions (IRFs) that show the effect of an unexpected change in, for stance, the change of money supply (ΔM2) on the change of the relative prices. We start this section presenting some comments about the Structural Vector Autoregression (SVAR). The Structural VAR (SVAR) can be represented by

$$AY_{t}=\alpha +\sum_{i=1}^{p}A_{i}Y_{t-i}+\varepsilon_{t}\text{ for }t=\;0:T,$$ (3)

If we assume that *A*_*0*_ is invertible then Eq (5) has a reduced form given by

$$Y_{t}\;=\;\beta \;+\;\sum_{i\;=\;1}^{p}B_{i}\;Y_{t-i}\;+\;u_{t}$$ (4)

with *u*_*t*_ ~ *N*(*0*,Σ)and $E(u_{t}u_{s}^{'})=0,\forall t\neq s$, where u_t_ is the reduced form residuals and *β* is a vector of constants. It is assumed that *ε*_*t*_ ~ *N*(0,*I*). The relation between models (3) and (4) is based on the following identities:

$$\beta =A^{-1}\alpha ,\;B_{i}=A^{-1}A_{i},\;u_{t}=A^{-1}\varepsilon_{t}\;and\;\sum =A^{-1}E(\varepsilon_{t}\varepsilon_{t}^{'})(A^{-1})'=A^{-1}(A^{-1})'.$$ (5)

Note that this representation does not allow identifying the effects of exogenous independent shocks onto the variables because the reduced form residuals are contemporaneously correlated (the Σ matrix is not diagonal). These shocks are primitive and exogenous forces, with no common causes, that affect the variables of the model. It is not possible to distinguish whose exogenous shocks affect the residual of which reduced form equation. It is possible to estimate the reduced form parameters *B* and Σ in Eq (5) consistently but, except for forecasting, they are not the parameters of interest. Without additional restrictions on *A* we cannot recover the structural form from the reduced form. This problems arise because Σ does not have enough estimated coefficients to recover an unrestricted *A* matrix. Therefore, we need to impose a number of restrictions that will allow us to identify and estimate *A*. This procedure is named identification.

The matrix *A* can be estimated using the information given by the covariance matrix of the reduced form. In general there are a large number of full rank matrices *A* that allow us to reproduce $\hat{\Sigma }$. That is, there are several conditional dependency and independency contemporaneous relations (“Markov kernels”) between the variables—given by different specifications in which some of the parameters in *A* are restricted to zero and others not—that allow us to reproduce the partial correlations observed for the reduced form residuals. It means, for instance, that $A_{}^{-1}{\left(A_{}^{-1}\right)}^{'}={\tilde{A}}_{}^{-1}{\left({\tilde{A}}_{}^{-1}\right)}^{'}$ in which $\tilde{A}$ is the Choleski decomposition for $\hat{\Sigma }$. In summary, there is no unique decomposition for $\hat{\Sigma }$.

The matrix *A* cannot have, together, a number of free parameters bigger than the number of free parameters in the symmetric matrix Σ. If n is the number of endogenous variables of the model then, to satisfy the order condition for identification of *A*, it is necessary that the number of free parameters to be estimated in *A* be no bigger than n(n-1)/2. When n is smaller than n(n-1)/2 the model is over-identified. There exists no simple general condition for local identification of the parameters of A. However, as has been shown by [59], a necessary and sufficient condition for local identification of any regular point in R^n^ is that the determinant of the information matrix be different from zero. In practice, evaluations of the determinant of the information matrix at some points, randomly chosen in the parameter space, is enough to establish the identification of a model.

In order to estimate the structural model it is necessary to identify a number of conditional independence relations (that is, parameters equal to zero in A) to satisfy the order condition for identification. Therefore, identifying *A* is equivalent to identifying the conditional distributions (“Markov Kernels”) of reduced form residuals. The identification procedure determines the order of causality among the endogenous variables of structural VAR. In accordance to [60] usually the identification is done using one of three approaches: a) applying Cholesky decomposition on the residuals covariance matrix Σ and implying a recursive order[54]; b) imposing some structural relation on matrix *A*[61,62]; or c) separating transitory from permanent shocks on primary impulses *ε*_*t*_ [63].

In this current research, we use Cholesky decomposition in the order

$$\Delta M2\to \Delta (P_{i}/P_{j})\to \Delta P\to \Delta I\to \Delta \tilde{Y}\to U$$

This means that the matrix *A* is triangular inferior and in accordance with this identification there exists a contemporaneous effect of an impulse of Δ*M2* in relative price Δ(*P*_*i*_/*P*_*j*_) but not the reverse. Also in according to Austrian theory of business cycle the effect on output comes from relative prices. In the end of chain we find the variables Δ*P*, Δ*I*, *U* and $\Delta \tilde{Y}$. Because the temporal dimension of our database is short, we decide to estimate the VAR excluding ΔY.

### Database

All data in this paper come from the economic database of Federal Reserve Bank of St. Louis (FRED) available at http://research.stlouisfed.org/fred2/. We use quarterly data from 1959:02 to 2013:02. Table 1 displays the description of variables. In this paper, we use the monetary aggregate M2 to represent the quantity of money. This broader concept is the measure of money supply commonly used in monetary studies.

**Table 1:** Description of aggregate variables (Frequency: Quarterly).

Series ID	Acronym	Title	Units
GDPPOT	*Y*_*P*_	Real Potential Gross Domestic Product	Billions of Chained 2005 Dollars
GDPC1	GDP	Real Gross Domestic Product	Billions of Chained 2009 Dollars
GPDIC96	Inv	Real Gross Private Domestic Investment	Billions of Chained 2009 Dollars
NROU	U	Natural Rate of Unemployment (Long-term)	Percent
GDPDEF	*P*_*GDP*_	Gross Domestic Product: Implicit Price Deflator	Index 2009 = 100
M2SL	M2	M2 Money Stock	Billions of Dollars
PPIACO	*P*_*i*_	Producer Price Index: All Commodities	Index 1982 = 100
CPIAUCSL	*P*_*j*_	Consumer Price Index for All Urban Consumers: All Items	Index 1982–84 = 100
CPITRNSL	CPITRNSL	Consumer Price Index for All Urban Consumers: Transportation	Index 1982–84 = 100
CPIMEDSL	CPIMEDSL	Consumer Price Index of All Urban Consumers: Medical Care	Index 1982–84 = 100

Source: Federal Reserve Bank of St. Louis: Federal Reserve Economic Data–FRED

In this context, M2 is M1 plus most savings accounts, money market accounts, retail money market mutual funds, and small denomination time deposits (certificates of deposit of under USD 100,000). M1: The total amount of M0 (cash/coin) outside of the private banking system plus the amount of demand deposits, travelers checks and other checkable deposits. M0: The total of all physical currency, including coinage. M0 = Federal Reserve Notes + US Notes + Coins. It is not relevant whether the currency is held inside or outside the private banking system as reserves.

## Econometric Results

This section presents the econometric results of the estimated model defined by Eqs (1) and (2). For that, we used estimates through generalized method of moments (GMM). As we noted in last section, we have four systems of equations, each one composed by two equations. Table 2 displays the regressions according to Eq 1, i.e., the models 1A, 2A, 3A and 4A. The results show that for any of these models all variables are statistically significant at the 1% level. The J-statistics, based on p-values higher than 0.90, do not provide evidence to reject the hypothesis of overidenfication. Hence, the model specification is not rejected. The Eq (1) was estimated a bit differently. We introduce the real output growth with 3 lags. The empirical results show that only the percentage change in the real output in the third lag (Y_t-3_) is statistically significant.

**Table 2:** Estimation Method: GMM (Eq 1—1959:2 to 2013:2).

Dependent variable: Δ(*P*_*i*_/*P*_*j*_)_*t*_ = (Producer Price Index: All Commodities/Consumer Price Index for All Urban Consumers: All Items)				
	Model 1A	Model 2A	Model 3A	Model 4A
Variables	Coefficient (S.E)	Coefficient (S.E)	Coefficient (S.E)	Coefficient (S.E)
Constant	0.005418* (0.000734)	0.005536* (0.001019)	0.005865* (0.001000)	0.006072* (0.000605)
Δ(*P*_*i*_/*P*_*j*_)_*t*−1_	0.389264* (0.009076)	0.391777* (0.010133)	0.398108* (0.009783)	0.388807* (0.005615)
Δ(*P*_*i*_/*P*_*j*_)_*t*−2_	-0.154510* (0.012432)	-0.164199* (0.014716)	-0.173615* (0.018148)	-0.150699* (0.007175)
Δ*M*_*t*_	-0.602219* (0.046343)	-0.600764* (0.064054)	-0.632170* (0.060963)	-0.632291* (0.042000)
ΔY_t-3_	0.259941* (0.026392)	0.244817* (0.030201)	0.270810* (0.024045)	0.250522* (0.026539)
*D(60*,*70)*	-0.020954* (0.003176)	-0.024295* (0.002921)	-0.026637* (0.003106)	-0.022493* (0.002589)
*D*(60,70)* Δ*M*	1.264765* (0.153779)	1.442976* (0.133276)	1.538495* (0.155278)	1.350944* (0.134785)
R-squared	0.102667	0.076849	0.059462	0.093553
J-Statistics	0.215983	0.182846	0.179790	0.219601
[p-value]	[0.995]	[0.995]	[0.995]	[0.995]
OBS	213	213	213	213

Source: Prepared by authors. Note1: * p-value ≤ 0.01; (SE) = Standard Error. Note2: Instruments—GDPDEF(-1to-5) CPITRNSL(-1to-5) CPIMEDSL(-1to-5) GDPPOT(-1to-5) CPIAUCSL(-1to-5) PPIACO (-1to-5).

The lags of the percentage change in the relative prices show opposite signs for all models. This contrast is necessary to control the seasonality pattern of inertia and dynamics present in this time series. The effects of the percentage change in the money supply and in the real GDP on the percentage change in the relative prices are negative and positive, respectively, for all models in Table 2. In this context, there is no empirical evidence that changes in relative prices are caused only by changes in real variables.

The coefficients of the dummy variable, *D*(60,70) and the interaction variable *D*(60,70)*(*M*) show negative and positive signs, respectively, for the specifications presented in Table 2. The results show that the effect of the percentage change in the money supply on the percentage change in relative prices is positive in the 1960s and 1970s. This means that in periods that the Fed was more tolerant of high inflation rates, the effect was higher.

In sum, the empirical results show that relative prices are not only affected by real variables, such as the percentage change in the real *GDP*, but also by nominal variables, such as the percentage change in the money supply.

Table 3 shows the estimates of the Models 1B, 2B, 3B and 4B according to Eq (2). The results shown by Models 1B to 4B indicate that all estimated coefficients are significant at 10% level, except Δ*M*_*t*_ and *D*(60,70)*Δ*M* in Eq 4B.

**Table 3:** Estimation Method: GMM (Eq 2—1959:2 to 2013:2).

Dependent variable: Deflator GDP (%) ΔPt (%)		Dependent variable: Real Investment (%) ΔIt (%)		Dependent variable: Natural Unemployment (%) U_t_ (%)		Dependent variable: Potential GDP (%) $\Delta {\tilde{Y}}_{t}$(%)	
Model 1B		Model 2B		Model 3B		Model 4B	
Variables	Coefficient (S.E.)	Variables	Coefficient (S.E.)	Variables	Coefficient (S.E.)	Variables	Coefficient (S.E.)
Constant	0.00029**(0.000121)	Constant	0.026687*(0.001559)	Constant	0.010128*(0.001357)	Constant	-3.61E-07(8.20E-06)
Δ*P*_*t*−1_	0.575034*(0.012085)	Δ*I*_*t*−1_	0.314183*(0.017844)	Δ*U*_*t*−1_	1.623866*(0.047124)	$\Delta {\tilde{Y}}_{t-1}$	1.925763*(0.008846)
Δ*P*_*t*−2_	0.314477*(0.012951)	Δ*I*_*t*−2_	0.02952***(0.016418)	Δ*U*_*t*−2_	-0.23778**(0.096240)	$\Delta {\tilde{Y}}_{t-2}$	-0.926537*(0.008762)
Δ(*P*_*i*_/*P*_*j*_)_*t*_	0.030810*(0.003256)	Δ(*P*_*i*_/*P*_*j*_)_*t*_	0.137611*(0.027890)	Δ*U*_*t*−3_	-0.387533*(0.049441)	Δ(*P*_*i*_/*P*_*j*_)	0.002603*(8.71E-05)
				Δ(*P*_*i*_/*P*_*j*_)_*t*_	-0.183967*(0.009370)		
Δ*M*_*t*_	0.0135***(0.007467)	Δ*M*_*t*_	-1.510148*(0.108372)	Δ*M*_*t*_	-0.159690*(0.031132)	Δ*M*_*t*_	0.000485(0.000334)
[D(60,70)*ΔM_t_]	0.057242*(0.004078)		0.587738*(0.099331)		0.068645**(0.028116)		-0.000151(0.000353)
R2	0.813910		0.038768		0.999702		0.993490

Source: Prepared by authors. Note1: * p-value ≤ 0.01; ** p-value ≤ 0.05; *** p-value ≤ 0.10; (SE) = Standard Error. Note2: Instruments—GDPDEF(-1to-5), CPITRNSL(-1to-5), CPIMEDSL(-1to-5), GDPPOT(-1to-5), CPIAUCSL(-1to-5), PPIACO (-1to-5).

The first system is represented by Model 1A in Table 2 and by model 1B in Table 3. Model 1B shows the direct effect of the percentage change in the relative prices on the inflation rate, with the estimated coefficient value of 0.030810. All the estimated coefficients of models 1A and 1B are significant at the 5% level, except the coefficient of change of money at model 1B, which it is marginally significant at level of 10%. However, the indirect effect shows that variations in the money supply affect relative prices (Model 1A), which in turn affect the rate of inflation (Model 1B). This means that a quarterly increase of 1% in ΔM (%) will negatively affect Δ(Pi/Pj) (%) by 0.602219%. Given that a 1% decrease in Pi/Pj (%) reduces inflation rate (π) by 0.030810%, the final effect of a 1% increase in M (%) will cause a quarterly decrease in π in 0.01855%. In turn, the final effect of a 1% increase in Yt-3 will cause a quarterly increase in π of 0.008%. Note that if the relative prices were defined as (Pj / Pi) instead of (Pi / Pj), the estimated coefficients of the change in relative prices would present opposite signs to those found in Tables 2 and 3.

The second system is represented by Model 2A in Table 2 and by model 2B in Table 3. Model 2B shows the effect of the percentage change in the relative prices on the variation of real investment level, with an estimated coefficient value of 0.137611. Nevertheless, the indirect effect shows that variations in the money supply affect changes in relative prices (Model 2A), which in turn affect the variation of real investment level (Model 2B). This means that a quarterly increase of 1% in ΔM2 (%) will negatively affect Δ(Pi/Pj) (%) in 0.600764%. Given that a 1% decrease in Δ(Pi/Pj) (%) reduces Δ*I* by 0.137611%, the final effect of a 1% increase in ΔM2 (%) will cause a quarterly decrease in Δ*I* of 0.08267%.

The third system is represented by Model 3A in Table 2 and by model 3B in Table 3. Model 3B shows the effect of the percentage change in relative prices on the natural unemployment rate, with an estimated coefficient value of -0.183967. Note that the indirect effect shows that variations in the money supply affect changes in relative prices (Model 3A), which in turn affect the natural rate of unemployment (Model 3B). This means that a quarterly increase of 1% in ΔM2 (%) will negatively affect Δ(Pi/Pj) (%) by 0.632170%. Given that a 1% decrease in Δ(Pi/Pj) (%) increases U (%) by 0.183967%, the final effect of a 1% increase in ΔM2 (%) will cause a quarterly increase in U (%) by 0.1163%.

Finally, the fourth system is represented by Model 4A in Table 2 and by model 4B in Table 3. Model 4B shows the effect of the percentage change in the relative prices on the percentage change in the real potential GDP, with an estimated coefficient value of 0.002603. The indirect effect shows that variations in the money supply affect changes in relative prices (Model 4A), which in turn affect the percentage change in the real potential output (Model 4B). This means that a quarterly increase of 1% in ΔM2 (%) will negatively affect Δ(Pi/Pj) (%) by 0.632291%. Given that a 1% decrease in Δ(Pi/Pj) (%) reduces $\Delta \tilde{Y}\;$ (%) by 0.002603%, the final effect of a 1% increase in ΔM2 (%) will cause a quarterly decrease in $\Delta \tilde{Y}\;$ (%) by 0.00164%.

In sum, the empirical results show that variations in the money supply affect relative prices. This result does not corroborate the assumption that changes in relative prices occur only by changes in real variables such as unemployment rate changes or technological shocks. Moreover, changes in relative prices, resulting from changes in the money supply, are transmission mechanisms of monetary policy because they indirectly affect real economic variables such as the variation of real investment, natural unemployment rate and variation of real potential output.

Although the results above are interesting from a theoretical and empirical perspective, the tests of weak instruments could not be evaluated due to the excessive number of regressors. Tables 4 and 5 below show a more parsimonious model that allows an assessment of the Stock-Yogo test. We use the same instruments for all estimates according to note 2 of Tables 2, 3, 4 and 5. In other words, we use the same instruments in every system of equations.

**Table 4:** Estimation Method: GMM (Eq 1—1959:2 to 2013:2).

Dependent variable: Δ(*P*_*i*_/*P*_*j*_)_*t*_ = (Producer Price Index: All commodities/Consumer Price Index for All Urban Consumers: All Items)				
	Model 1C	Model 2C	Model 3C	Model 4C
Variables	Coefficient (S.E.)	Coefficient (S.E.)	Coefficient (S.E.)	Coefficient (S.E.)
Constant	-0.003007*(0.000186)	-0.002665*(0.000208)	-0.002741*(0.000221)	-0.002625*(0.000182)
Δ(*P*_*i*_/*P*_*j*_)_*t*−1_	0.355756*(0.004185)	0.351685*(0.006875)	0.354188*(0.008572)	0.350549*(0.004517)
Δ*Y*_*t*−3_	0.199093*(0.012875)	0.144066*(0.022130)	0.170447*(0.015166)	0.145544*(0.015496)
D(60,70)*Δ*M*_*t*_	0.122809*(0.012244)	0.115116*(0.022077)	0.103094*(0.027689)	0.107281*(0.010787)
R2	0.089502	0.095355	0.094136	0.095981
J-Statistics	0.226680	0.190744	0.180391	0.226678
[p-value]	[0.995]	[0.995]	[0.995]	[0.995]
OBS	213	213	213	213
Weak Instrument Diagnostics				
Stock-Yogo test	Cragg-Donald F-Statistic	20.63452	Critical values (relative bias) 5%	20.37

Source: Prepared by authors. Note1: * p-value ≤ 0.01; (SE) = Standard Error. Note2: Instruments—GDPDEF(-1to-5), CPITRNSL(-1to-5), CPIMEDSL(-1to-5), GDPPOT(-1to-5), CPIAUCSL(-1to-5), PPIACO (-1to-5).

**Table 5:** Estimation Method: GMM (Eq 2—1959:2 to 2013:2).

Dependent variable: Deflator GDP (%) ΔPt (%)		Dependent variable: Real Investment (%) ΔIt (%)		Dependent variable: Natural Unemployment (%) U_t_ (%)		Dependent variable: Potential GDP (%) $\Delta {\tilde{Y}}_{t}$(%)	
Model 1D		Model 2D		Model 3D		Model 4D	
Variables	Coefficient (S.E.)	Variables	Coefficient (S.E.)	Variables	Coefficient (S.E.)	Variables	Coefficient (S.E.)
Constant	0.000369*(6.83E-05)	Constant	0.025890*(0.00254)	Constant	0.010178*(0.00117)	Constant	-7.67E-06(9.13E-06)
Δ*P*_*t*−1_	0.576500*(0.01125)	Δ*I*_*t*−1_	0.342861*(0.01246)	Δ*U*_*t*−1_	1.597926*(0.04391)	$\Delta {\tilde{Y}}_{t-1}$	1.923987*(0.00758)
Δ*P*_*t*−2_	0.305459*(0.01068)	Δ*I*_*t*−2_	-0.13825*(0.03395)	Δ*U*_*t*−2_	-0.1845**(0.08951)	$\Delta {\tilde{Y}}_{t-2}$	-0.92372*(0.00749)
Δ(*P*_*i*_/*P*_*j*_)_*t*_	0.028506*(0.00224)	Δ(*P*_*i*_/*P*_*j*_)_*t*_	0.148112*(0.05730)	Δ*U*_*t*−3_	-0.41486*(0.04570)	Δ(*P*_*i*_/*P*_*j*_)	0.002489*(0.00014)
				Δ(*P*_*i*_/*P*_*j*_)_*t*_	-0.171819*(0.008011)		
Δ*M*_*t*_	0.013495*(0.00480)	Δ*M*_*t*_	-1.371633*(0.160348)	Δ*M*_*t*_	-0.130399*(0.017195)	Δ*M*_*t*_	0.00075***(0.000470)
[D(60,70)*ΔM_t_]	0.050811*(0.00328)		0.472362*(0.099308)		0.068884*(0.026883)		-0.000815*(0.000270)
R2	0.816871		0.040097		0.999705		0.993503

Source: Prepared by authors. Note1: * p-value ≤ 0.01; ** p-value ≤ 0.05; *** p-value ≤ 0.10; (SE) = Standard Error. Note2: Instruments—GDPDEF(-1to-5), CPITRNSL(-1to-5), CPIMEDSL(-1to-5), GDPPOT(-1to-5), CPIAUCSL(-1to-5), PPIACO (-1to-5).

The F statistic indicates evidence for the rejection of the null hypothesis of weak instruments, as shown in Table 4. The value of Cragg-Donald F-statistic is 20.63452 and the Stock-Yogo critical values TSLS at the 5% level of significance is 20.27.

Tables 2 and 4 present results for Eq (1). The models estimated in Table 4 differ by omitting the dummy of monetary regimes D (60,70) and the growth rate of the money supply, ΔM, making it the most parsimonious. However, the two variables are represented by the interaction variable, which is the product resulting from them, D(60, 70)*ΔM. The other difference is that the estimates in Table 4 fulfill the need for the instrumental variables to be "good instruments" to be relevant and valid. It is also worth noting that the explanatory variables in common between Tables 2 and 4 present the estimated coefficients statistically significant at the 1% level; they also have the same signals except for the constant term.

Considering the results of the estimates of Eq (2) presented in Tables 3 and 5, we can see that there are some differences between the results. The estimated coefficient of change of money of Table 3 (Model 1B) is statistically significant at 10% level; meanwhile the same estimated coefficient of Table 5 (Model 1D) is statistically significant at 1% level. Besides, the estimated coefficients of ΔM% and D(60, 70)*ΔM of Table 3 (Model 4B) is not statistically significant at 10% level; meanwhile in Table 5 these same variables are statistically significant (Model 4D). These estimated coefficients show the direct impact of change of money on *V*_*t*._ We thank the referee for suggesting introducing the direct effect of change of money on *V*_*t*_ to avoid a biased estimator of *δ*_1_ in Eq 2.

In the next section, we will provide a more accurate discussion of the empirical results presented in Tables 4 and 5.

### Structural VAR (SVAR) analysis

**Fig 1:**
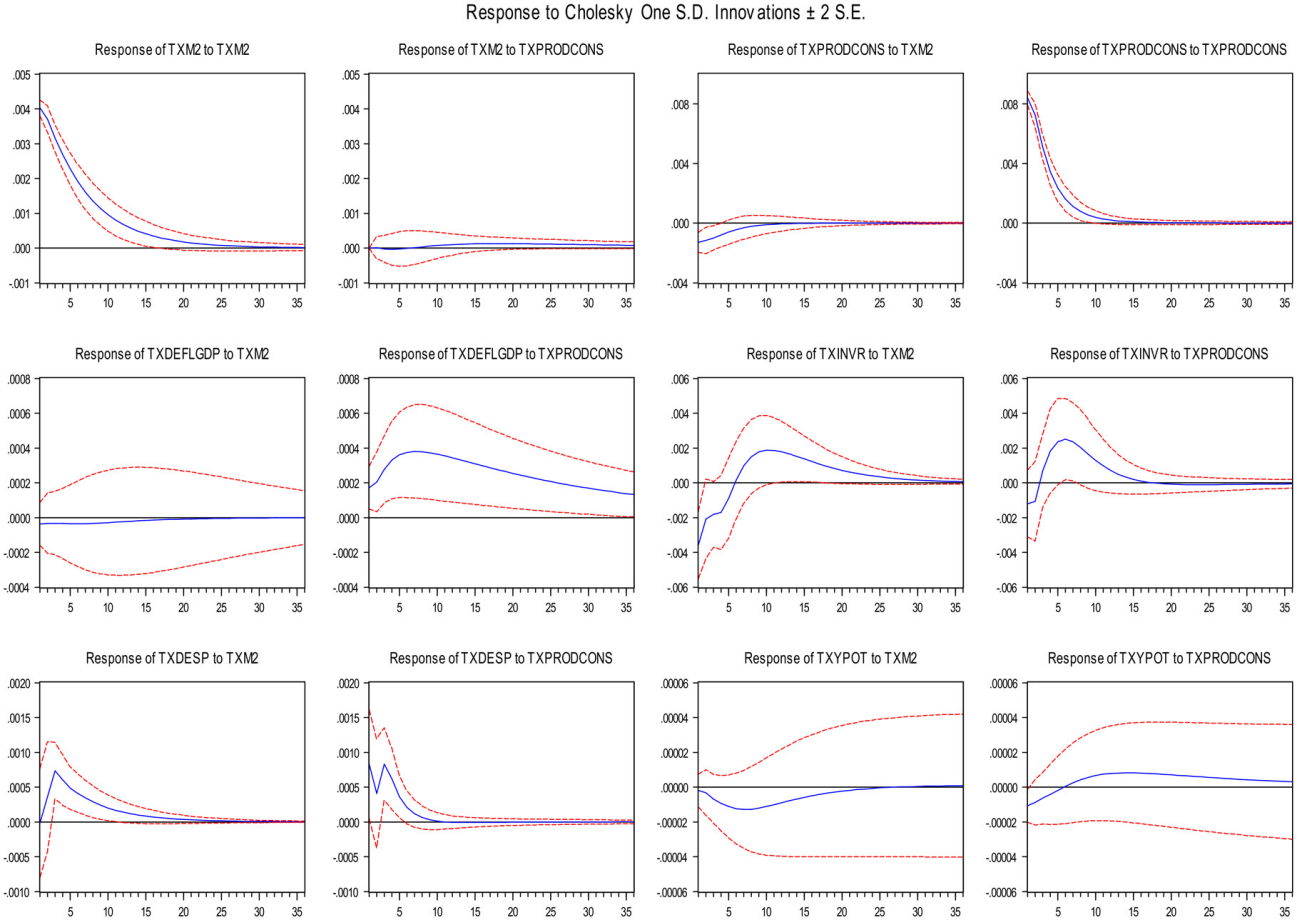
Impulse Response Functions

The VAR was estimated using quarterly data from 1959Q2 to 2013Q2 with two lags following the SC (Schwarz) information criterion, FPE (final prediction error) and HQ (Hannan-Quinn) information criteria. We also perform the unit roots in the series of variables and one does not detect any problem of nonstationarity in the data. Fig 1 displays impulse response functions. For economy, we do not present all the IRF’s, but only the effect of a shock of the M2 and the relative price in the time horizon of nine years.

We now compare the results derived from the IRFs to GMM’s results. In Table 2 we can see that for all cases the effect of a change in M on a change in relative price is negative. In Fig 1 an unexpected change in ΔM (TXM2) has negative impact on the variation of relative prices *P*_*i*_*/P*_*j*_ [Δ(*P*_*i*_*/P*_*j*_) = TXPRODCONS] although this effect disappears around twelve quarterlies ahead. Considering the results from Table 3, the IRF´s of a change of relative prices on a change on GDP deflator (ΔP = TXDEFLGDP), a change on real investment (ΔI = TXINVR) and a change on the potential output ($\Delta \tilde{Y}\;$ = TXYPOT) are in accordance with the results of Table 3. However, the IRF of TXPRODCONS on natural unemployment rate (U = Txdesp) is positive and remains in a short horizon of nine quarterlies ahead. In sum, the shocks of ΔM on a change of relative prices produce effect as well as the shocks of Δ(*P*_*i*_*/P*_*j*_) on the other variables of interest (ΔP, ΔI, $\Delta \tilde{Y}\;$ and *U*).

## Analysis

The quantity theory of money assumes that relative price changes are caused only by real variables, such as changes in aggregate output, in employment or any changes that alter production costs, pressure from unions, monopoly power of firms or scarcity of goods, among others. The empirical results of this study show that changes in relative prices stem not only from changes in real variables but also from changes in the money supply. If monetary policy generates changes in relative prices then we can infer that it also alters the allocation of production factors and alters the goods and services produced, resulting in income redistribution.

Regardless of whether money affects relative prices, most economists believe that money is not neutral in the short term. However, if money affects relative prices, policymakers have a major complicating factor to manage monetary policy. They will have to monitor the spread of money and credit throughout all sectors of the economy. They will have to map how different sectors of the economy will be affected differently. The simple fact that there are changes in relative prices will generate an income redistribution within the economy, which may result in an increase or decrease in income concentration. This effect is evident when we notice empirical evidence showing that changes in relative prices resulting from changes in the money supply affect the inflation rate, considering the monetary regime of greater tolerance for inflation in the 1960s and 1970s.

Before reviewing the indirect effects of monetary policy via changes in relative prices on inflation rate, change of real investment and long-term variables, we will comment on one of the basic tenets of QTM, namely the dichotomy relative price/absolute prices.

This dichotomy is linked to the fact that changes in real variables cause changes in relative prices, while changes in absolute prices are attributed to monetary causes. In other words, given the supply of money, the velocity of money and the level of transactions of goods, changes induced by a real shock in the relative prices produce compensatory measures in other prices, leaving unchanged the level of absolute prices.

This strong assumption should startle readers because nothing guarantees that this compensatory effect will occur. The empirical results of this study do not support the postulate of TQM on the dichotomy between relative and absolute prices in a monetary regime of greater tolerance for inflation.

The above-mentioned result initially shows a short-term effect of monetary expansion and changes in relative prices on the variation of investment. According to Austrian economists, when the central bank or commercial banks expand money and credit, the new money thus created is initially spent on specific goods and services. The demands for these products go up in relation to the demands of others, which increase their prices relative to other prices. As the new money spreads throughout the economy, demand for other goods increase and hence other prices will increase as well. Wealth and income redistribution is in favor of those who received the new currency at the beginning of the process at the expense of those who only receive it in the later stages. Therefore, there are two types of relative price changes. The first is the redistribution of income from late to first receivers of the new money, which occurs during the inflationary process. The second is the permanent changes in wealth and income that continue to occur even after the money has already spread throughout the economy. This permanent change in the redistribution of income and wealth shows the non-neutrality of money in the long run.

Another relevant point concerning the work of the Austrian school is the development of the theory of the business cycle [30,32]. Mises shows that the creation of expansionary credit and deposits, without corresponding effective savings caused by a banking system based on a fractional reserve ratio directed by a central bank, not only generates cyclical and uncontrolled growth of the money supply but also in the creation of lending at interest rates that are artificially low. This inevitably generates a "flare" artificial and unsustainable production process. Thus, the economy tends to become excessively capital intensive. The expansionary process resulting in inflation tends to revert. Entrepreneurs will liquidate the investments wrongly induced by artificial credit expansion [30,31,32].

Empirical results (Model 2D, Table 5) show that the change of real investment responds positively to changes in relative prices. The variation of investment could respond negatively to changes in relative prices if the index was defined as consumer price index/consumer price index. This result suggests that when the index of producer prices grows relatively more than the consumer price index, the rate of return on investments will increase. On the other hand, an increase in the consumer price index reduces workers’ compensation, ceteris paribus. In this sense, it also suggests that there is a transfer of income among economic agents.

Empirical results (Models 2C and 2D) still show some indirect effects on the variation of investment through changes in relative prices. Based on model 2C we observe that a greater variation from the real product directly results in greater variation in relative prices and indirectly a higher variation of investment. Still, based on the 2C model we observe that in the 1960s and 1970s further monetary expansion directly resulted in greater variation in relative prices and indirectly in higher change of investment.

They argue that excessive expansion of the money supply and consequent credit expansion distorts the system of incentives or signals received by investors due to the change in relative prices. Therefore, investors would not invest in a situation where the market credit does not suffer government interventions to monetize the economy. These resource allocations in investment projects with high return rates artificially explain an initial economic boom followed by a subsequent reduction in the level of economic activity, which can be seen in Tables 2 and 3 (models 2A and 2B), because the estimated coefficient of variation of the supply of money is negative. This behavior of economic agents can explain the economic cycle.

Below we examine the effects of a change in the money supply through changes in relative prices on real long run variables (Table 5). The empirical results for the various models estimated show that the natural rate of unemployment responds to three lags of that variable with positive and negative signs and responds negatively to changes in relative prices. This means that increases in relative price changes adversely affect the natural rate of unemployment. This result shows that when the index of producer prices grows relatively more than the consumer price index, such a move will result in lower natural unemployment rate.

Regarding the potential output, we see that it responds positively to changes in relative prices. This result shows that increases in relative prices positively affect the growth rate of potential output. We observe that a greater variation of the supply of money in the 1960s and 1970s directly resulted in greater changes in relative prices and indirectly in a higher rate of potential GDP. See Tables 4 and 5. In this context, expansionary monetary policies generate higher rates of inflation, reduction in natural unemployment rate and increased variation in potential output.

## Final Remarks

This article evaluates the direct impact of the growth of the money supply on the change in relative prices, as well as the indirect impact of changes in relative prices on other variables of interest, considering the period (1960s and 1970s) in which the Federal Reserve Bank was more tolerant of inflation. For theoretical reasons, we use the ratio of the producer price index and consumer price index to represent relative prices. In other words, we investigate the direct impact of monetary policy on relative prices through a quarterly database of the U.S. economy for the period 1959:2 to 2013:2. Moreover, we also investigate the indirect impact of monetary policy via changes in relative prices on the rate of inflation, on the variation of real investment and on long-term variables such as the natural unemployment rate and real potential GDP changes.

Based on estimates of equations obtained through GMM results we show that variations in the money supply in the 1960s and 1970s directly affected relative prices, which in turn indirectly affected the inflation rate, variation of real investment, the natural unemployment rate and the variation of real potential output.

The empirical results of this study show that changes in relative prices stem not only from changes in real variables but also from changes in the money supply. If monetary policy generates changes in relative prices then we can infer that it also alters the allocation of production factors and alters the goods and services produced, resulting in income redistribution. In this context, the relevant achievement of our study is that money is not neutral in a non-traditional sense because a change in the money supply distorts the relative prices and consequently the allocation of resources in an economy.

Furthermore, there exist a serious implication that must be considered in the issue of monetary policy transmission mechanisms, which occurs through changes in relative prices. It would be difficult for policymakers to predict the outcome that interactions of real and monetary shocks have on relative prices and, hence, on the variables of interest, such as inflation rate, variation of real investment, natural unemployment rate and real output growth.
